# Interventions for Preventing Cardiotoxicity in Breast Cancer Patients Receiving Trastuzumab: A Systemic Review and Bayesian Network Meta-Analysis

**DOI:** 10.3389/fphar.2021.718086

**Published:** 2021-08-18

**Authors:** Xinyi Li, Ziyang Wu, Xin Du, Yibo Wu, Xiaohui Xie, Luwen Shi

**Affiliations:** ^1^ Department of Pharmacy Administration and Clinical Pharmacy, School of Pharmaceutical Sciences, Peking University, Beijing, China; ^2^ Institute for Drug Evaluation, Peking University Health Science Center, Beijing, China

**Keywords:** trastuzumab, cardiotoxicity, short duration of trastuzumab, cardioprotective drugs, meta-analysis

## Abstract

**Background:** Trastuzumab is associated with the risk of cardiotoxicity. Here, we aim to explore interventions for preventing trastuzumab-related cardiotoxic effects in breast cancer patients.

**Methods:** A systematic review was performed including trials of breast cancer patients with intervention to prevent cardiotoxicity of trastuzumab. Trials were searched through databases including PubMed, Embase, and Cochrane Library.

**Results:** Eight RCTs were included. Five trials reported the outcomes of short-duration interventions, including 6-month and 9-week durations, and only 9-week treatment has a significant difference from the 12-month group (OR 0.38; 95% CI 0.18–0.83) using cardiotoxicity as the outcome. However, 6-month treatment turned out to yield less occurrence of trastuzumab discontinuation (OR 0.32; 95% CI 0.24–0.42). Three trials reported interventions of cardioprotective drugs, and there is no significant difference shown in any cardioprotective group compared with placebo (cardiotoxicity outcome: angiotensin-converting enzyme inhibitor: OR 0.48; 95% CI 0.057–2.3; angiotensin receptor blocker: OR 1.3; 95% CI 0.12–14; β-blocker: OR 0.48; 95% CI 0.057–2.3; trastuzumab interruption outcome: angiotensin-converting enzyme inhibitor: OR 0.45; 95% CI 0.12–1.3; angiotensin receptor blocker: OR 0.87; 95% CI 0.15–4.8; β-blocker: OR 0.41; 95% CI 0.11–1.2).

**Conclusion:** Only the 9-week group has a significant difference from the 12-month group using cardiotoxicity as the outcome. And 6-month treatment turned out to yield less occurrence of trastuzumab discontinuation. The use of cardioprotective drugs failed to prevent trastuzumab-related cardiotoxic effects in breast cancer patients.

## Introduction

Female breast cancer has leapt to be the most commonly diagnosed cancer surpassing lung cancer, with an estimated 2.3 million new cases in 2020 (Sung et al., 2021). With the development of detection, targeted therapy, and supportive care, the survival rates of breast cancer have continued to improve, and the overall five-year breast cancer survival rate has reached 98% for stage Ⅰ in the United States (DeSantis et al., 2019). More and more people have got cured from breast cancer, but there is evidence that they have got an increased risk of cardiovascular disease and death from cardiovascular disease because of the adverse effects of anticancer therapy (Zamorano et al., 2016; Blaes and Konety, 2021).

Trastuzumab, an antibody targeting human epidermal growth factor receptor 2 (HER2), delivered with chemotherapy for patients with HER2-positive breast cancer has made a great progress in the therapy of metastatic and adjuvant settings (Piccart-Gebhart et al., 2005; Romond et al., 2005; Perez et al., 2014; Cameron et al., 2017). However, trastuzumab is associated with the risk of cancer treatment–related cardiotoxicity (Procter et al., 2010; de Azambuja et al., 2014). There is a great need for effective interventions to prevent or limit the cardiotoxic effects of trastuzumab. Previous meta-analysis has shown that shorter treatment durations decreased the risk of severe cardiac toxicity compared to 12 months of trastuzumab (Eiger et al., 2020). However, there is lack of comparison between different treatment durations of trastuzumab. Besides, recent studies demonstrated that cardioprotective drugs might be likely to reduce the cardiotoxicity effect caused by the 12-month duration of trastuzumab in breast cancer patients (Guglin et al., 2019). Whether these drugs can confer cardioprotective effects in trastuzumab-treated patients is still unclear.

To address this question, we performed a systematic review including randomized controlled trials (RCTs) of interventions for preventing cardiotoxicity in breast cancer patients receiving trastuzumab. A network meta-analysis was performed to analyze the cardiotoxicity effects of various therapies by integrating all available direct and indirect evidence.

## Methods

### Search Strategy

This study was carried out following the Preferred Reporting Items for Systematic Reviews and Meta-Analyses (PRISMA) criteria. To perform a systematic review of the published literature, two independent investigators identified the relevant studies through databases including Embase, PubMed, and Cochrane Library up to February 1, 2021. The electronic search was performed using the following search keywords: (“breast cancer” OR “breast neoplasm” OR “breast tumor” OR “breast tumour” OR “breast carcinoma”) and (“trastuzumab” OR “Herceptin”). The searches were limited to randomized controlled trials (RCTs).

The studies including breast cancer patients who were given any intervention to prevent cardiotoxicity of trastuzumab were eligible. Exclusion criteria were as follows: 1) studies coming from conference abstracts, 2) studies without relevant data, 3) studies without full text, 4) ongoing trials, and 5) trial protocols.

### Data Extraction and Outcome

The data on study name, design, sample size, background medication therapy, trial intervention, median age, and cardiac outcomes were extracted by two independent investigators.

We accepted two outcome definitions (Abdel-Qadir et al., 2017): 1) cardiotoxicity: all types of cardiac dysfunctions in individual studies or a clinically relevant decline in left ventricular ejection fraction (LVEF), utilizing the cut-off used in individual studies, and 2) interruptions in trastuzumab therapy: failing to complete the required trastuzumab treatment duration due to any given clinical cardiac events.

### Quality Assessment

The Cochrane Collaboration’s risk of bias tool was used to perform the quality assessment of the included studies, including six items: sequence generation, allocation concealment, blinding of outcome data, incomplete outcome data, selective outcome reporting, and free from other bias, which were ranked to have high, unclear, and low risk of bias for each trial.

### Statistical Methods

R (v4.0.3) and the gemtc package (v0.8-8) were used to perform our Bayesian network meta-analysis. A random-effects model was conservatively applied. ORs and corresponding 95% credible intervals (CrIs) served as the indices. The heterogeneity was assessed by the “mtc.anohe” command in the “gemtc” package. The heterogeneity between studies was assessed as high if I^2^ > 50%; on the contrary, the heterogeneity between studies was assessed as low. The trace and density plots were used to evaluate the convergence of models. A *p*-value (two-sided) less than 0.05 was considered statistically significant. The hierarchy of treatments was determined by calculating the rank probabilities. In addition to network meta-analysis, a pairwise meta-analysis concerning two managements was performed by Stata (v15.1).

## Results

### Study Characteristics

**FIGURE 1:**
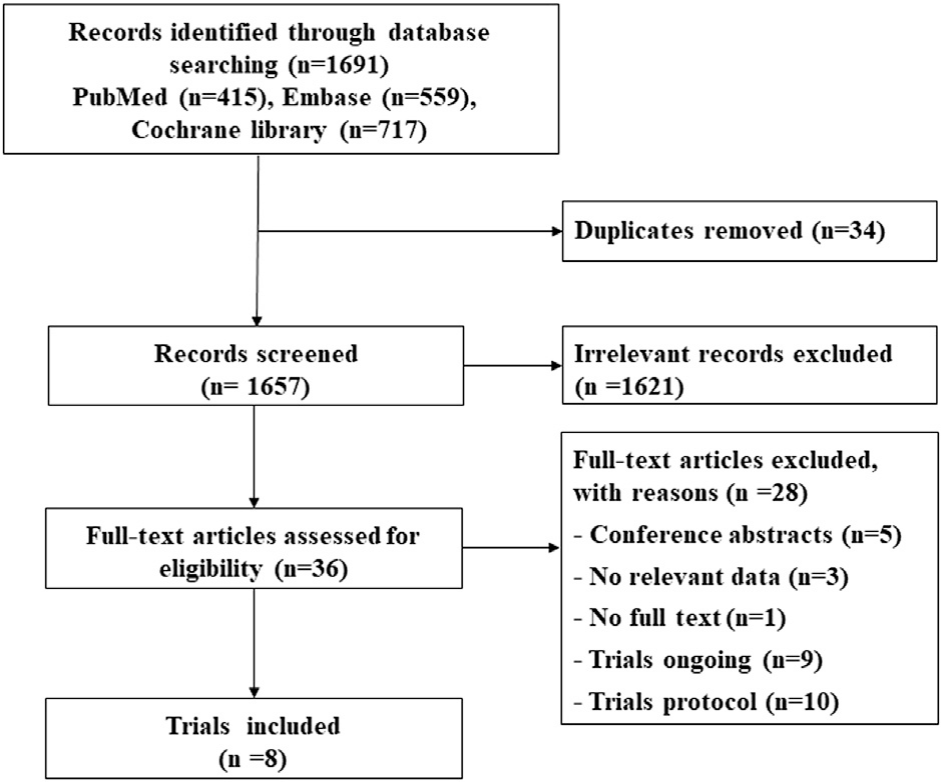
Flow diagram of the selection process.

**FIGURE 2:**
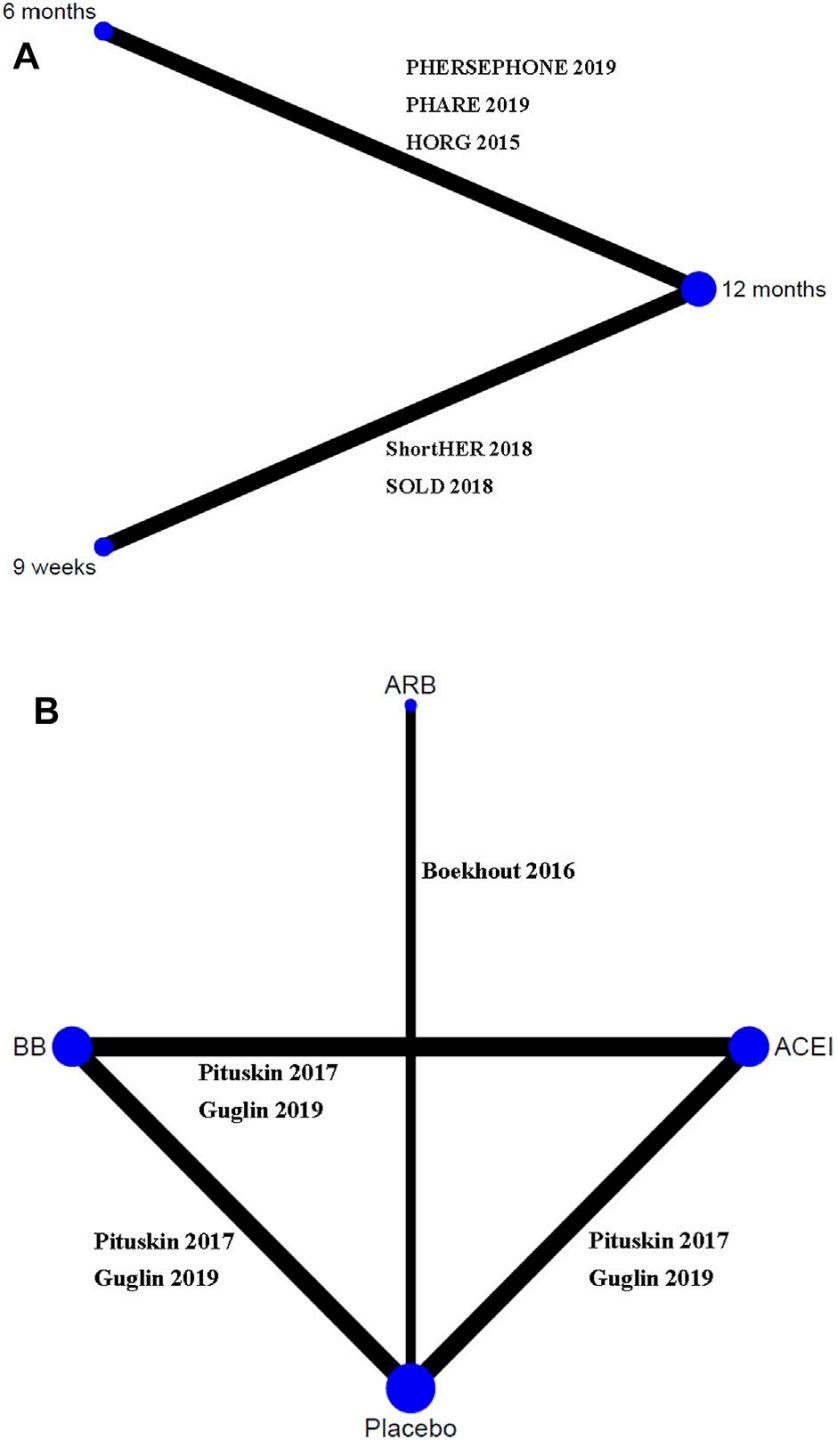
Network evidence plots of interventions for preventing cardiotoxicity in breast cancer patients receiving trastuzumab. **(A)** Short duration of trastuzumab using cardiotoxicity as the outcome; **(B)** cardioprotective drug intervention.

A total of 1,691 citations were identified through electronic searches, and eight RCTs were selected for our study (Figure 1) (Mavroudis et al., 2015; Boekhout et al., 2016; Pituskin et al., 2017; Conte et al., 2018; Joensuu et al., 2018; Earl et al., 2019; Guglin et al., 2019; Pivot et al., 2019). Of these, five studies assessed whether fewer cardiac events can be achieved with reduced treatment duration, including a 6-month vs a 12-month schedule (Mavroudis et al., 2015; Earl et al., 2019; Pivot et al., 2019) and a 9-week vs a 12-month schedule (Conte et al., 2018; Joensuu et al., 2018). Other three studies assessed the medical intervention for preventing the cardiotoxicity effect of trastuzumab (Boekhout et al., 2016; Pituskin et al., 2017; Guglin et al., 2019), including the intervention of β-blocker (BB), angiotensin receptor blocker (ARB), or angiotensin-converting enzyme inhibitor (ACEI) (Table 1). The treatment network is shown in Figure 2. Three studies reported 6 months of trastuzumab vs 12 months, and two studies reported 9 weeks of trastuzumab vs 12 months for reduced treatment intervention (Figure 2A). In addition, two studies reported placebo vs BB, placebo vs ACEI, and ACEI vs BB, and one study reported placebo vs ARB for medical intervention (Figure 2B).

**TABLE 1:** Study characteristics of eligible trials.

						Cardiac outcomes	
Study	Design	Sample size	Background medication therapy	Trial intervention	Median/mean age (years)	Cardiotoxicity	Interruptions in trastuzumab therapy
**Short duration of trastuzumab**							
PHERSEPHONE, 2019	RCT, phase III	4,088	Anthracycline/taxane + H	12 months	56	224/1968	146/1894
				6 months	56	155/1994	61/1939
PHARE, 2019	RCT, phase III	3,380	Anthracycline/taxane + H	12 months	54	111/1690	49/1690
				6 months	55	67/1690	0/1690
HORG, 2015	RCT, phase III	481	FEC → D + H	12 months	54	NR^a^	0/241
				6 months	56		2/240
ShortHER, 2018	RCT, phase III	1,253	AC/EC → T/D+H	12 months	55	82/627	NR
			D + H → FEC	9 weeks	55	27/626	
SOLD, 2018	RCT, phase III	2,174	D + H → FEC ± H	12 months	56	42/1089	NR
				9 weeks	56	22/1085	
**Cardioprotective drugs**							
Pituskin (2017)	RCT, phase II	94	AC/FEC → D+H	Placebo	30	6/30	9/30
			D + carboplatin + H	ACEI	33	1/33	3/33
				BB	31	1/31	3/31
Boekhout (2016)	RCT, phase III	206	Anthracycline/taxane + H	Placebo	50	16/103	9/103
				ARB	50	20/103	8/103
Guglin (2019)	RCT, phase II	468	40.4% exposed to anthracycline	Placebo	51.11	46/143	40/152
				BB	51.58	43/149	24/156
				ACEI	50.58	45/149	27/156

Abbreviations: AC = doxorubicin/cyclophosphamide; EC = epirubicin/cyclophosphamide; FEC = 5-FU/epidoxorubicin/cyclophosphamide; T = paclitaxel; D = docetaxel; H = Herceptin; NR = not reported.

a Cardiac toxicity did not differ between the two arms.

### Risk of Bias Assessment

All eight studies were considered to be of high quality with a low risk of selection, attrition, and reporting biases. There is high risk of blinding in the studies of short-duration intervention given their open-label design (Supplementary Material S1).

### Short Duration of Trastuzumab

**FIGURE 3:**
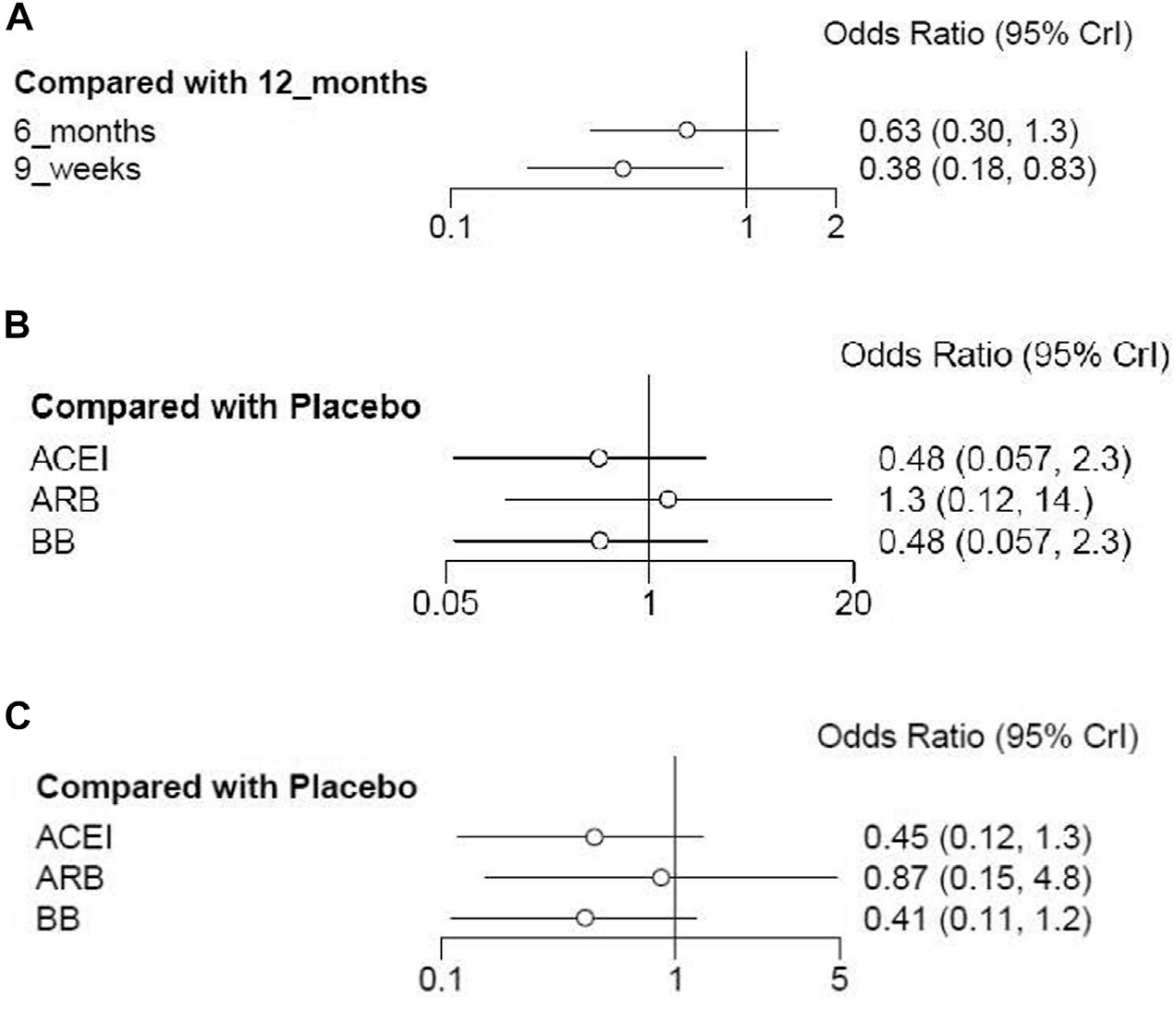
Forest plots of the effect of interventions for reducing cardiotoxicity in breast cancer patients receiving trastuzumab. **(A)** Short duration of trastuzumab using cardiotoxicity as the outcome; **(B)** cardioprotective drug intervention using cardiotoxicity as the outcome; **(C)** cardioprotective drug intervention using interruptions in trastuzumab therapy as the outcome.

**FIGURE 4:**
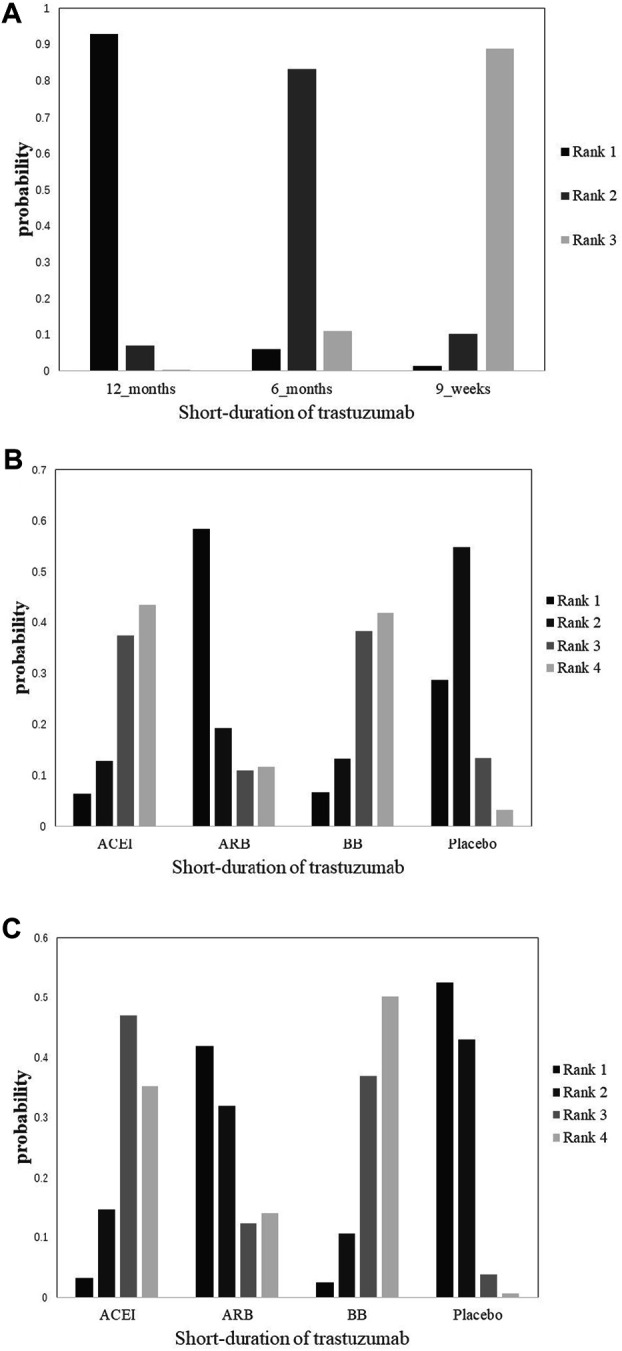
Rank plots of the probability for cardiotoxicity in breast cancer patients receiving trastuzumab. **(A)** Short duration of trastuzumab using cardiotoxicity as the outcome; **(B)** cardioprotective drug intervention using cardiotoxicity as the outcome; **(C)** cardioprotective drug intervention using interruptions in trastuzumab therapy as the outcome.

Cardiotoxicity: Four studies with 10,769 patients reported cardiac events of short duration vs 12-month treatment. Only treatment with the 9-week group has a significant difference from the 12-month group (OR 0.38; 95% CI 0.18–0.83; *p* = 0.015; Figure 3A). No significant difference was seen between the 6-month group and the 12-month group for the occurrence of cardiac events (OR 0.63; 95% CI 0.30–1.3; *p* = 0.21; Figure 3A). Based on treatment ranking, cardiotoxicity increased with the treatment time. The 12-week trastuzumab treatment ranked first for cardiotoxicity, followed by 6-month treatment and 9-week treatment. The detailed ranking results are shown in Figure 4A.

**FIGURE 5:**
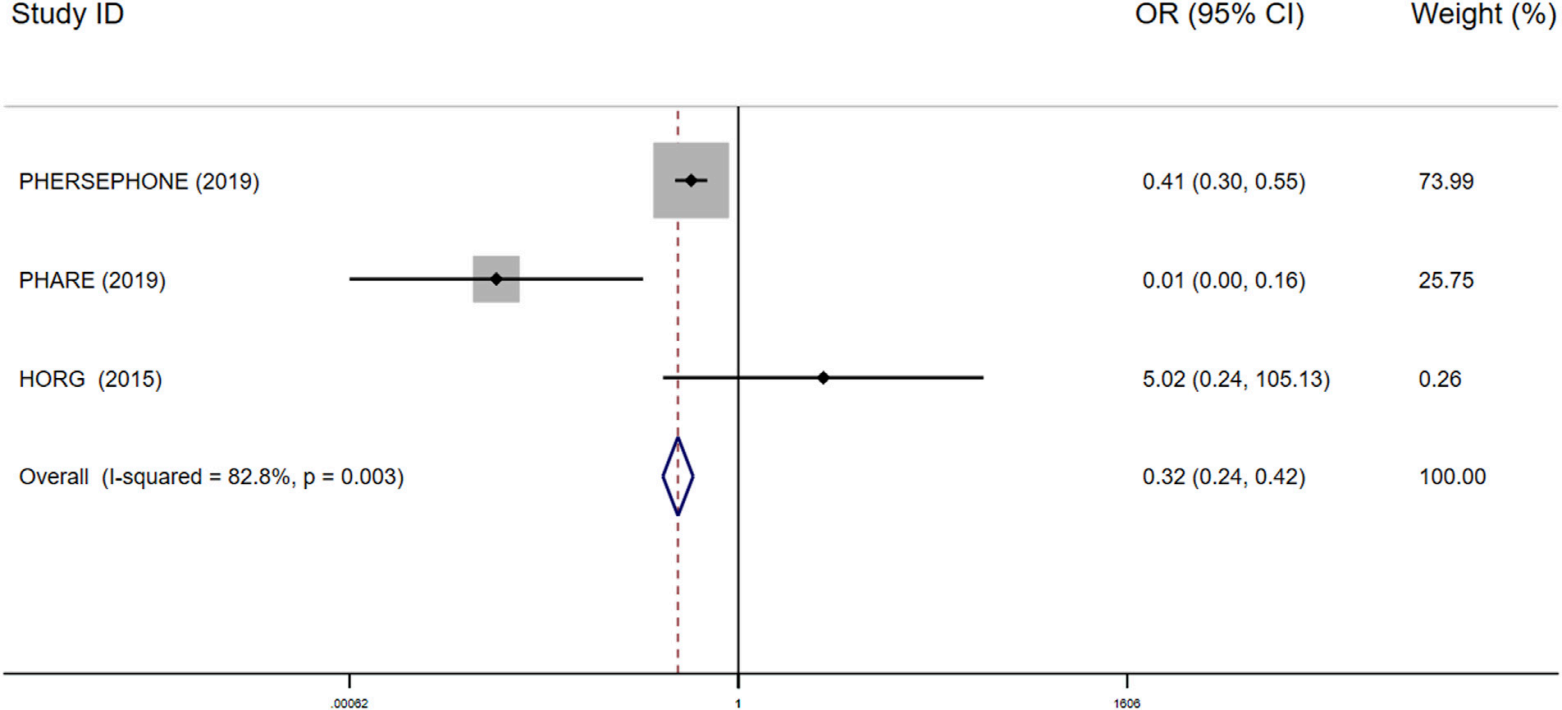
Forest plot of the effect of short-duration interventions for decreasing occurrence of trastuzumab discontinuation in breast cancer patients receiving trastuzumab.

Interruptions in trastuzumab therapy: Only three studies consisting of 7,694 patients reported the interruptions in the 6-month treatment group vs the 12-month treatment group. Compared with the 12-month treatment group, the 6-month group turned out to yield less occurrence of trastuzumab discontinuation (OR 0.32; 95% CI 0.24–0.42; I^2^ = 82.8%; P<0.001; Figure 5).

### Cardioprotective Drugs

Cardiotoxicity: Three studies with 764 patients were included in the analysis. Compared with placebo, no significant advantage was seen in any cardioprotective group (ACEI: OR 0.48; 95% CI 0.057–2.3; *p* = 0.28; ARB: OR 1.3; 95% CI 0.12–14; *p* = 0.83; BB: OR 0.48; 95% CI 0.057–2.3; *p* = 0.28; Figure 3B). Based on treatment ranking, ARB and placebo exhibited the highest probability of yielding more occurrence of cardiac events, followed by BB and ACEI. The detailed ranking results are shown in Figure 4B.

Interruptions in trastuzumab therapy: Three studies with 764 patients reported the interruption events. No significant difference was shown in any cardioprotective group compared with placebo (ACEI: OR 0.45; 95% CI 0.12–1.3; *p* = 0.13; ARB: OR 0.87; 95% CI 0.15–4.8; *p* = 0.85; BB: OR 0.41; 95% CI 0.11–1.2; *p* = 0.10; Figure 3C). Based on treatment ranking, placebo exhibited the highest probability of yielding more occurrence of interruptions in trastuzumab therapy, followed by ARB, ACEI, and BB. The detailed ranking results are shown in Figure 4C.

### Convergence and Heterogeneity Analyses

The single chain fluctuation cannot be recognized through the naked eye, and the density map was normally distributed, reflecting good convergence in this analysis. The heterogeneity was discovered through the heterogeneity analysis in the 9-week vs 12-month groups using cardiotoxicity as the outcome (I^2^ = 58.3545%), 6-month vs 12-month groups using interruptions in trastuzumab therapy as the outcome (I^2^ = 82.8%; Figure 5), the ACEI group vs the placebo group using cardiotoxicity as the outcome (I^2^ = 80.45342%), and the BB group vs the placebo group (I^2^ = 79.03769%) using cardiotoxicity as the outcome; there is no other heterogeneity seen in the overall pooled analysis. The full results of the convergence and heterogeneity analyses are illustrated in Supplementary Material S2.

## Discussion

The HER2-targeting agent trastuzumab plays an important role in the treatment of HER2-overexpressing breast cancer. However, there is evidence that trastuzumab is associated with the risk of cardiotoxicity without the clear mechanism. Previous findings demonstrate that the oxidative balance is likely disrupted due to trastuzumab because the HER2 signaling pathway plays a part in the moderation of oxidative stress in cardiac myocytes (Harbeck et al., 2011; Zagar et al., 2016). Recognizing the effects of trastuzumab on the heart, we explore the interventions to prevent or decrease cardiotoxicity.

Currently, the optimal treatment duration of trastuzumab in HER2-positive patients is still controversial because the duration was chosen casually to conduct the clinical trials (Pinto et al., 2013; Cameron et al., 2017). New trials have been conducted to compare the short-duration therapy with 12-month therapy. However, 12-month adjuvant trastuzumab is still the standard therapy although it has cardiac toxic effects according to the current evidence (Chen et al., 2019; Inno et al., 2019; Niraula and Gyawali, 2019). To explore cardiotoxicity between different therapies, a meta-analysis was conducted, which showed shorter treatment durations decreased the occurrence of cardiac events (Eiger et al., 2020). In our study, only 9-week duration therapy has a significant difference of cardiotoxicity from 12-month trastuzumab although the results show that cardiotoxicity is decreased with the shorter treatment. Besides, E219817, a trial comparing treatment of the 12-month group with 12-week treatment, has reported that 12-month treatment with trastuzumab did not significantly increase the occurrence of cardiac events with heart failure as the outcome. However, we found that the 6-month group turned out to yield less occurrence of trastuzumab discontinuation compared with 12 months of trastuzumab. In this way, the regimen should be made according to the risk factors of cardiotoxicity and financial situation of patients, in order to achieve the balance of efficacy, safety, and economy.

Cardioprotective drugs might be a choice to prevent cardiotoxicity of trastuzumab for breast cancer patients, such as ARB, ACEI, and BB, since pharmacological interventions have been proven effective in the prevention of anthracycline-related toxic effects (Kalam and Marwick, 2013; Abdel-Qadir et al., 2017; Caspani et al., 2020). Unfortunately, the results did not support the hypothesis that cardioprotective drugs (ARB, ACEI, and BB) could decrease cardiotoxicity or the occurrence of trastuzumab discontinuation during trastuzumab treatment. In addition, the retrospective studies have assessed the cardioprotective effect of statins in patients receiving trastuzumab therapy (Calvillo-Argüelles et al., 2019; Abdel-Qadir et al., 2021). One study showed that the exposed statins can decrease the risk of cardiotoxicity (Calvillo-Argüelles et al., 2019), while another did not support this conclusion (Abdel-Qadir et al., 2021). More trials are needed in this area to assess whether cardioprotective drugs can be effective to prevent the cardiotoxicity effect of trastuzumab and which one will be the most effective.

In addition to the interventions above, physical exercise is recommended to prevent cardiotoxicity in breast cancer patients receiving trastuzumab. A clinical trial is ongoing with the purpose of evaluating the impact of 3 months of exercise intervention on myocardial function and especially on the risk of cardiotoxicity (Jacquinot et al., 2017). The final result is worth the wait.

Besides, the anthracyclines, which are also the important treatment component in breast cancer, can induce myocardial oxidative stress and cause cardiotoxicity. The patients who were treated with both trastuzumab and anthracyclines might have a greater risk of cardiotoxicity. However, most of the clinical trials that we included did not report data for an exploratory subgroup analysis of anthracycline and non-anthracycline groups. There is only one clinical trial with the subgroups of anthracycline and non-anthracycline cohorts, which illustrated that ACEI and BB could prevent cardiotoxicity in patients receiving both trastuzumab and anthracyclines instead of those receiving trastuzumab without anthracyclines.

To our best knowledge, this study is the first systemic review and meta-analysis to summarize the prevention of trastuzumab-mediated cardiotoxicity. In this study, we included two kinds of interventions including the short duration of trastuzumab and add-on therapy of cardioprotective drugs, which gave clinically important advice in this area.

However, there are some limitations in this systemic review in light of the small dataset size and limited trial data. At first, only five eligible trials were included for comparing short-duration treatment with 12 months of trastuzumab treatment and three trials were included for comparing cardioprotective drugs with placebo. Moreover, the different definitions of cardiac events in different studies consulted in heterogeneity. In addition, we did not include multiple factors in the cardiotoxicity analysis of trastuzumab because of the unavailability of the data.

In conclusion, the use of cardioprotective drugs failed to prevent trastuzumab-related cardiotoxic effects in breast cancer patients. Compared to 12 months of trastuzumab, the evidence only supports that 9-week treatment decreases the occurrence of cardiac events. In contrast, 6 months of trastuzumab is associated with less occurrence of interruptions in trastuzumab therapy compared with 12 months of trastuzumab.

## Data Availability

The original contributions presented in the study are included in the article/Supplementary Material, and further inquiries can be directed to the corresponding author.
