# Supplementary material for: Interventions for Preventing Cardiotoxicity in Breast Cancer Patients Receiving Trastuzumab: A Systemic Review and Bayesian Network Meta-Analysis
Source: Front Pharmacol. 2021 Aug 18;12:718086. doi: 10.3389/fphar.2021.718086 (PMC8416464; doi:10.3389/fphar.2021.718086)
Supplement: Supplementary file 2 [file DataSheet1.docx]

***Results of convergence and heterogeneity analysis***

**1. Results of convergence analysis**

**1.1 Short-duration**

**Short-duration of trastuzumab group using cardiotoxicity as outcome**


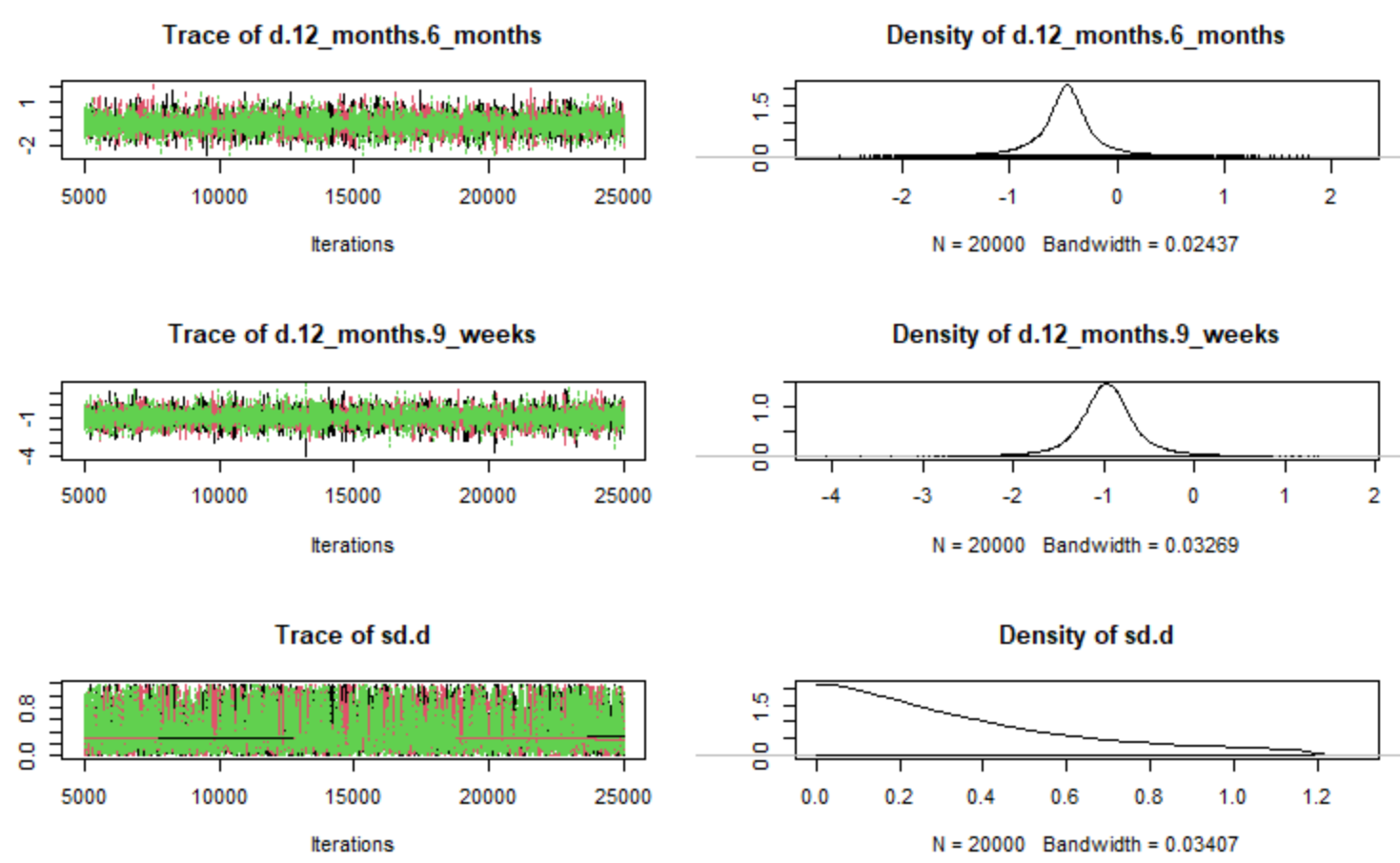


Supplementary Figure 1. Trace plot and density plot for short-duration of trastuzumab group using cardiotoxicity as outcome

**1.2 Cardioprotective drugs**

**Cardioprotective drugs group using cardiotoxicity as outcome**


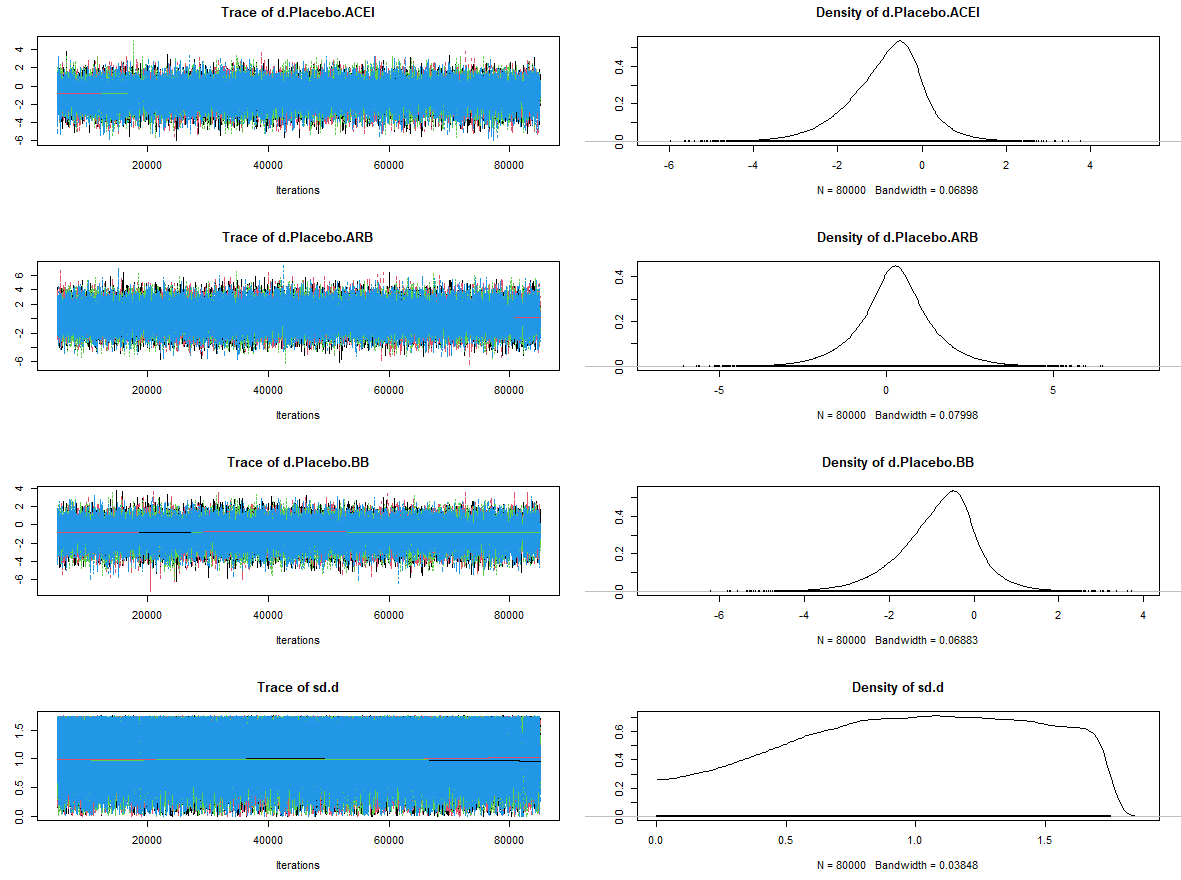


Supplementary Figure 2. Trace plot and density plot of cardioprotective drugs group using cardiotoxicity as outcome

**Cardioprotective drugs group using interruptions in trastuzumab therapy as outcome**


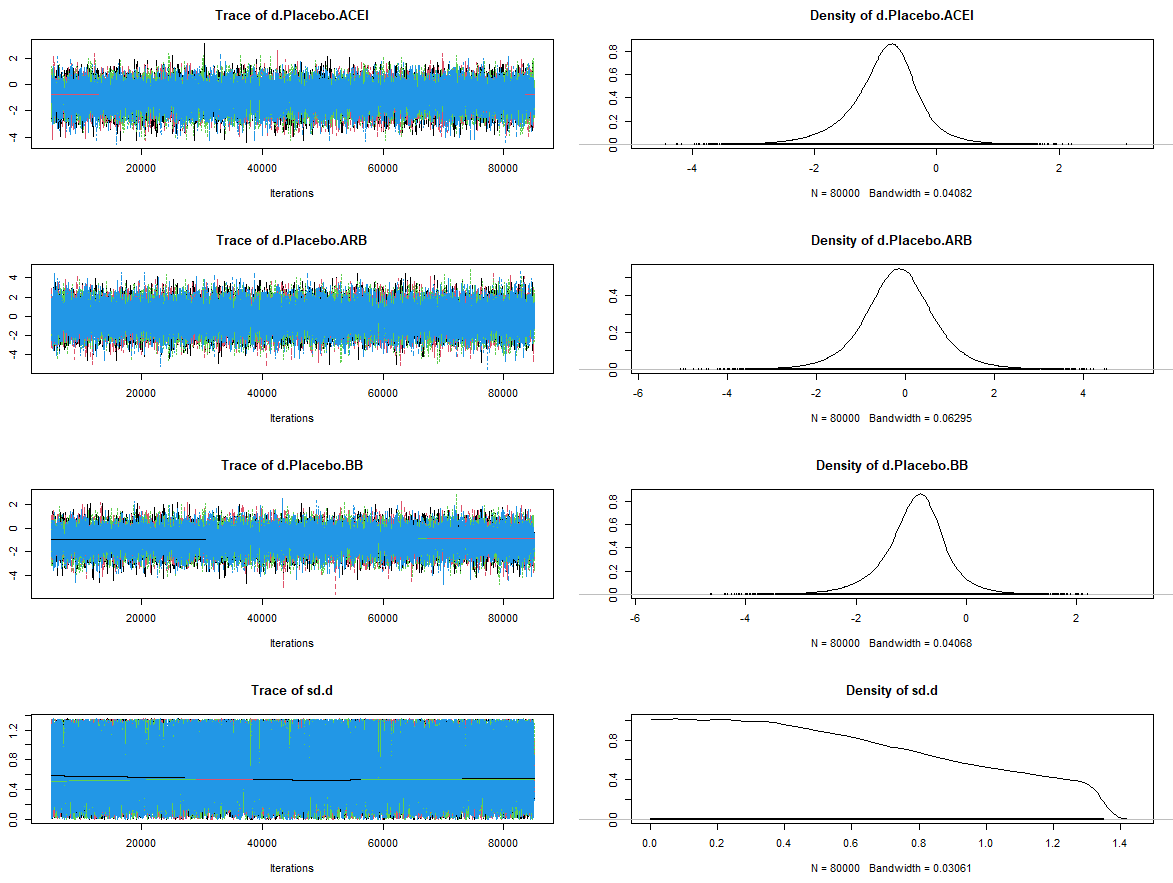


Supplementary Figure 3. Trace plot and density plot of cardioprotective drugs group using interruptions in trastuzumab therapy as outcome

**2. Results of heterogeneity** **analysis**

**2.1 Short-duration**

**Short-duration of trastuzumab group using cardiotoxicity as outcome**

Analysis of heterogeneity

=========================

Per-comparison I-squared:

-------------------------

|  | t1 | t2 | i2.pair | i2.cons | incons.p |
| --- | --- | --- | --- | --- | --- |
| 1 | 12_months | 6_months | 0.00000 | 0.00000 | NA |
| 2 | 12_months | 9_weeks | 58.32719 | 58.3545 | NA |

Global I-squared:

-------------------------

i2.pair i2.cons

1 27.08036 26.95222

**2.2 Cardioprotective drugs**

**Cardioprotective drugs group using cardiotoxicity as outcome**

Analysis of heterogeneity

=========================

Per-comparison I-squared:

-------------------------

|  | t1 | t2 | i2.pair | i2.cons | incons.p |
| --- | --- | --- | --- | --- | --- |
| 1 | ACEI | BB | 0.00000 | 0.00000 | NA |
| 2 | ACEI | Placebo | 79.34766 | 80.45342 | NA |
| 3 | ARB | Placebo | NA | NA | NA |
| 4 | BB | Placebo | 77.35157 | 79.03769 | NA |

Global I-squared:

-------------------------

i2.pair i2.cons

1 67.59652 59.55049

**Cardioprotective drugs group using interruptions in trastuzumab therapy as outcome**

Analysis of heterogeneity

=========================

Per-comparison I-squared:

-------------------------

|  | t1 | t2 | i2.pair | i2.cons | incons.p |
| --- | --- | --- | --- | --- | --- |
| 1 | ACEI | BB | 0.00000 | 0.00000 | NA |
| 2 | ACEI | Placebo | 26.49885 | 27.6681 | NA |
| 3 | ARB | Placebo | NA | NA | NA |
| 4 | BB | Placebo | 0.00000 | 0.00000 | NA |

Global I-squared:

-------------------------

i2.pair i2.cons

1 0 0
